# Draft Genome Sequence of *Altererythrobacter marensis* DSM 21428^T^, Isolated from Seawater

**DOI:** 10.1128/genomeA.01607-15

**Published:** 2016-02-04

**Authors:** Hong Cheng, Yue-Hong Wu, Ying-Yi Huo, Chun-Sheng Wang, Xue-Wei Xu

**Affiliations:** Laboratory of Marine Ecosystem and Biogeochemistry, Second Institute of Oceanography, State Oceanic Administration, Hangzhou, People’s Republic of China

## Abstract

*Altererythrobacter marensis* DSM 21428^T^ was isolated from seawater collected around Mara Island, South Korea. The genomic characteristics of this strain support its potential for alkane-related compound degradation. *A. marensis* DSM 21428^T^ has potential applications in bioremediation projects concerning offshore petroleum spill prevention and response.

## GENOME ANNOUNCEMENT

*Altererythrobacter marensis* DSM 21428^T^ is a Gram-negative and strictly aerobic marine bacterium belonging to the family *Erythrobacteraceae*. The strain was isolated from seawater around the coast of Mara Island, Jeju, South Korea (33.1° N, 126.18° E). Phylogenetic analysis revealed *A. marensis* to be closely related to *A. epoxidivorans*, which is the type species of the genus *Altererythrobacter* (1) and demonstrates petroleum degradation activity (2). Here, we report the draft genome sequence of *A. marensis* DSM 21428^T^ and discuss its petroleum degradation ability inferred from functional annotation.

Genomic DNA was isolated with the AxyPrep bacterial genomic DNA miniprep kit (Corning Life Sciences, USA). High-throughput sequencing was carried out on an Illumina HiSeq 2000 platform (Novogene Bioinformatics Technology Co., Ltd., Beijing) with a 500-bp insert size paired-end library. Sequencing generated 804 M of clean data with ~277-fold genome coverage. Reads were assembled *de novo* into contigs using SOAPdenovo (3) v2.0.1 and Abyss (4) v1.5.2. The assembly k-mer was tested from *k* = 36 to 64 for seeking the optimal value of *k* = 62 using Abyss. We used MUMmer (5) to estimate assembly quality. Gene prediction, tRNAs, and functional annotation were performed with PROKKA package v1.11 (6) as well as the RAST server online (7). Based on positions of open reading frames (ORFs) obtained from the system, the predicted ORFs were blastp (ncbi-blast-2.2.31+) against cluster of orthologous group (COG) (8) databases for orthologous clusters. Signal peptides and transmembrane helices were predicted using SignalP v4.1 (9) and TMHMM v2.0 (10). Additionally, tRNAs and rRNAs were confirmed using RNAmmer v1.2 (11) and tRNAscan-SE v1.21 (12).

The draft genome sequence of strain DSM 21428^T^ yields 14 contigs with an *N*_50_ of 915,391 bp and a total assembly length of 2,902,055 bp (G+C content 64.66%). It encodes 2,745 ORFs, 45 tRNAs, 1 transfer-messenger RNA (tmRNA), and 1 5S-23S-16S rRNA gene operon. A total of 2,159 ORFs were assigned to COGs. The numbers of signal peptides and transmembrane helices found in protein-coding genes were 322 and 583, respectively.

Alkane degradation and aromatic hydrocarbon cleavage are important processes in petroleum metabolism (13). The genome of strain DSM 21428^T^ encodes one cytochrome P450 alkane hydroxylase used in aerobic alkane digesting. It also contains enzymes which can be used in aromatic ring cleavage, including one aromatic hydrocarbon utilization transcriptional regulator *Cat*R and two dienelactone hydrolase family proteins. In other genomes of *Altererythrobacter* members, we found several related proteins involved in petroleum degradation. *A. epoxidivorans* (CP012669) contains cytochrome P450 hydroxylase (ALE17367) and cytochrome P450 alkane hydroxylase (ALE17700); *A. atlanticus* (CP011452) (14) encodes two dienelactone hydrolase family proteins (AKH41555 and AKH42951) and one maleylacetate reductase (AKH42916). Results of the comparative annotation revealed that not only *A. marensis* but also its closely related species can be used in alkane bioremediation. In addition, some *Erythrobacteraceae* strains contain various bacteriochlorophylls (BChls) as photosynthetic pigments for adapting to low-nutrition conditions. In this study, however, strain DSM 21428^T^ was not observed to contain BChl *a* (15), and Bchl *a* synthesis-related genes were not found in the genome sequence. This study will supply genome data for the genus *Altererythrobacter* and may illustrate adaption mechanisms of this group in the marine environment.

### Nucleotide sequence accession numbers.

The draft genome sequence of *A. marensis* strain DSM 21428^T^ has been deposited at DDBJ/EMBL/GenBank under the accession number LMVG00000000. The version described in this paper is version LMVG00000000.1
